# Antibacterial Activity and Antibiotic-Enhancing Effects of Honeybee Venom against Methicillin-Resistant *Staphylococcus aureus*

**DOI:** 10.3390/molecules21010079

**Published:** 2016-01-12

**Authors:** Sang Mi Han, Joung Min Kim, In Pyo Hong, Soon Ok Woo, Se Gun Kim, He Rye Jang, Sok Cheon Pak

**Affiliations:** 1 Rural Development Administration, National Academy of Agricultural Science, Wanju, Chonbuk 55365, Korea; jmyellow80@naver.com (J.M.K.); iphong20@korea.kr (I.P.H.); wooso1@korea.kr (S.O.W.); kimsegun@korea.kr (S.G.K.); tddas@naver.com (H.R.J.); 2 School of Biomedical Sciences, Charles Sturt University, Bathurst 2795, Australia; spak@csu.edu.au

**Keywords:** bee venom, MRSA, antibacterial effect, antibiotic effects, *atl*

## Abstract

Methicillin-resistant *Staphylococcus aureus* (MRSA), along with other antibiotic resistant bacteria, has become a significant social and clinical problem. There is thus an urgent need to develop naturally bioactive compounds as alternatives to the few antibiotics that remain effective. Here we assessed the *in vitro* activities of bee venom (BV), alone or in combination with ampicillin, penicillin, gentamicin or vancomycin, on growth of MRSA strains. The antimicrobial activity of BV against MRSA strains was investigated using minimum inhibitory concentrations (MIC), minimum bactericidal concentrations (MBC) and a time-kill assay. Expression of *atl* which encodes murein hydrolase, a peptidoglycan-degrading enzyme involved in cell separation, was measured by reverse transcription-polymerase chain reaction. The MICs of BV were 0.085 µg/mL and 0.11 µg/mL against MRSA CCARM 3366 and MRSA CCARM 3708, respectively. The MBC of BV against MRSA 3366 was 0.106 µg/mL and that against MRSA 3708 was 0.14 µg/mL. The bactericidal activity of BV corresponded to a decrease of at least 3 log CFU/g cells. The combination of BV with ampicillin or penicillin yielded an inhibitory concentration index ranging from 0.631 to 1.002, indicating a partial and indifferent synergistic effect. Compared to ampicillin or penicillin, both MRSA strains were more susceptible to the combination of BV with gentamicin or vancomycin. The expression of *atl* gene was increased in MRSA 3366 treated with BV. These results suggest that BV exhibited antibacterial activity and antibiotic-enhancing effects against MRSA strains. The *atl* gene was increased in MRSA exposed to BV, suggesting that cell division was interrupted. BV warrants further investigation as a natural antimicrobial agent and synergist of antibiotic activity.

## 1. Introduction

Since 1959 when methicillin was introduced to treat infections caused by penicillin-resistant *Staphylococcus aureus*, the incidence of methicillin resistance among staphylococcal strains has rapidly increased [1]. Due to widespread methicillin use, methicillin-resistant *Staphylococcus aureus* (MRSA) has become a major clinical problem globally [1]. MRSA infections are difficult to treat because of their multidrug-resistance properties, including resistance to β-lactams as well as several other classes of antibiotics [2,3]. It is known that antibiotic-induced killing of *Staphylococcus aureus* involves a novel regulator of murein hydrolase activity [4]. Murein hydrolases have been shown to be related to the susceptibility of bacteria to antibiotics. The most prominent murein hydrolase in the pathogenicity of *Staphylococcus aureus* is *atl* gene for its role in cell separation.

Bee venom (BV) from honeybee (*Apis mellifera* L.) has long been used as a complementary medicine to treat an array of conditions [5,6]. Pure BV is generally obtained by electric stunning using a BV collector without causing harm to the honeybees, removing impurities from the collected BV and lyophilizing the final product. BV is comprised of a number of bioactive substances such as melittin, apamin, adolapin and mast cell degranulating peptide [7]. In addition, it contains biologically active amines (histamine, epinephrine) and a few non-peptide components including lipids, carbohydrates and free amino acids [8]. In recent years, BV has been added as a cosmetic ingredient in anti- photoaging products based on previously published results [9]. BV is also known to be involved in antimicrobial and anti-inflammatory action against acne-inducing bacteria [10].

In the present study, we investigated further the antimicrobial activity of BV and the synergistic effects of BV in combination with ampicillin, penicillin, gentamicin or vancomycin against MRSA strains. To investigate whether interrupted cell division was due to defective cell separation, the effect of BV on the activity of murein hydrolases was determined based on the expression of *atl* measured by reverse transcription-polymerase chain reaction (RT-PCR).

## 2. Results and Discussion

### 2.1. Effects of BV on MRSA

The antimicrobial effects of BV on human pathogenic bacteria, including clinical isolates of antibiotic-resistant bacteria, were investigated and described as minimum inhibitory concentrations (MIC) and minimum bactericidal concentrations (MBC). MIC indicates the inhibitory potential of BV while MBC shows the killing potential of BV on clinically isolated MRSA strains. The MIC and MBC values of BV against MRSA CCARM 3366 and MRSA CCARM 3708 are shown in Table 1. As expected, slightly higher BV MBCs relative to the MIC were observed in our study. These findings indicate that BV inhibited the growth of these MRSA strains at relatively low concentrations.

**Table 1:** Minimum inhibitory concentration and minimum bactericidal concentration of BV against *S. aureus* 3366 and 3708.

MRSA Strains	MIC (µg/mL)	MBC (µg/mL)
CCARM 3366	0.085 ± 0.003	0.106 ± 0.006
CCARM 3708	0.11 ± 0.001	0.14 ± 0.009

### 2.2. Time-Kill Studies

**Figure 1:**
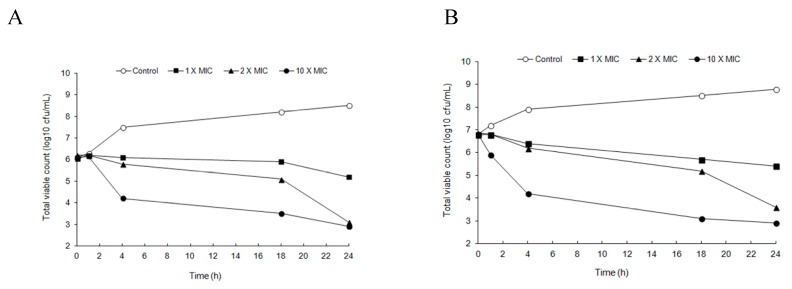
Time-kill curve showing the *in vitro* bactericidal effect of BV on Methicillin-resistant *Staphylococcus aureus* MRSA 3366 (**A**) and MRSA 3708 (**B**).

The time-kill curves of BV against MRSA strains are shown in Figure 1. BV activity was concentration-dependent, with increasing concentrations resulting in a progressive reduction of CFU. All concentrations exhibited antibacterial activity against both MRSA CCARM 3366 and MRSA CCARM 3708 over time, with the two highest concentrations achieving a reduction in bacterial count of a least 6 log units after 24 h. After more than 4 h of exposure, BV concentrations of 0.17 and 0.85 µg/mL (corresponding to 2 × MIC and 10 × MIC, respectively) exhibited bacteriostatic activity against MRSA CCARM 3366 based on viable cell counts (Figure 1A). In addition, BV concentrations of 0.17 and 0.85 µg/mL inhibited the growth of MRSA CCARM 3708 over a 24 h period (Figure 1B). After more than 4 h of exposure, BV concentrations of 0.11, 0.22 and 1.1 µg/mL (corresponding to 1 × MIC, 2 × MIC and 10 × MIC, respectively) exhibited bacteriostatic activity against MRSA CCARM 3708 based on the viable cell counts. A BV concentration of 1.1 µg/mL (corresponding to 10 × MIC) inhibited the growth of MRSA CCARM 3708 over an 18 h period.

### 2.3. Synergistic Effects of BV on MRSA

Ampicillin and penicillin as antimicrobial agents are basically ineffective on MRSA strains. We evaluated the possible synergistic effects of BV with antibiotics against MRSA strains. In the test results, the FIC of BV in combination with ampicillin or penicillin ranged from 0.001 to 1 μg/mL and 0.002 to 1 μg/mL against MRSA CCARM 3366 and MRSA CCARM 3708, respectively (Table 2). BV induced an increase in the activity of both ampicillin and penicillin. BV had partial synergistic effects with penicillin on both MRSA strains. On the other hand, BV had indifferent synergistic effects with ampicillin on either MRSA strain. It is noteworthy that both gentamicin and vancomycin enhanced the bactericidal activity of BV against both MRSA strains.

**Table 2:** Synergistic effects of BV with ampicillin or penicillin against Methicillin-resistant *Staphylococcus aureus* MRSA 3366 and 3708.

Strains	Agent	MIC		FIC (µg/mL)	FIC Index ^a^	Outcome
		Alone	Combination			
CCARM 3366	Ampicillin BV	435	1.06	0.002	1.002	indifferent
		0.085	0.085	1.00		
	Penicillin BV	680	1.33	0.001	0.631	partial synergy
		0.085	0.054	0.63		
	Gentamicin BV	0.5	0.025	0.05	0.14	synergy
		0.085	0.008	0.09		
	Vancomycin BV	2	0.55	0.27	0.48	synergy
		0.085	0.018	0.21		
CCARM 3708	Ampicillin BV	540	1.33	0.002	1.002	indifferent
		0.11	0.11	1.00		
	Penicillin BV	851	1.75	0.002	0.772	partial synergy
		0.11	0.085	0.77		
	Gentamicin BV	0.44	0.028	0.063	0.373	synergy
		0.11	0.035	0.31		
	Vancomycin BV	1.7	0.70	0.41	0.72	partial synergy
		0.11	0.035	0.31		

^a^ FIC index was interpreted as synergy at ≤0.5, partial synergy at >0.5 but <1.0, indifferent at >1.0 and <4.0, and antagonistic when values were ≥4.0.

### 2.4. Determination of atl Gene Expression

**Figure 2:**
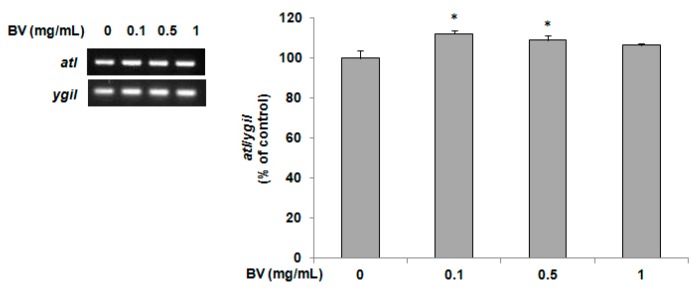
Reverse transcription-polymerase chain reaction for *atl* mRNA expression. The results show that BV treatment increases *atl* mRNA production in Methicillin-resistant *Staphylococcus aureus* MRSA 3366. Data are presented as mean ± SD values of three independent experiments. *: significant differences (*p* < 0.05) with an increase in mRNA expression relative to the control.

The *atl* gene as a regulator of murein hydrolases promotes autolysis and extracellular DNA release to facilitate biofilm formation in *S. aureus*. Compared with untreated MRSA, exposure of MRSA to BV led to increased levels of *atl* expression (*p* < 0.05, Figure 2).

### 2.5. Discussion

Due to the increasing number of MRSA strains exhibiting resistance to multiple antibiotics, there is an urgent need to develop new antibiotics [11]. Natural products such as BV are potential candidates to address this need. Repetitive chemical acupuncture therapy with BV was able to produce a robust analgesic effect on chronic neuropathic pain [12]. BV has been reported to have anti-cancer activities. Cancerous tumor growth was hampered by BV through induction of apoptosis in lung cancer cells, breast cancer cells, hepatocellular carcinoma cells and prostate cancer cells [13,14]. Recently BV has also been evaluated for antiaging and antibacterial functions making it ideal for use in skin care and cosmetic preparation. We reported skin photoprotective action of BV through reduction of protein levels of matrix metalloproteinases which are main contributors to photoaging processes [9]. Furthermore, BV augmented wound healing with concomitant inhibition of cytokines associated with fibrosis, which resulted in decreased wound size and increasing epithelial proliferation in a mouse full-thickness excision wound model [15]. We previously reported anti-inflammatory properties of BV which were demonstrated either by inhibiting inducible nitric oxide synthase (iNOS) and tumor necrosis factor-α expression, or by the regulation of NO generation that was dependent on nuclear factor kappa B/activator protein-1 through down-regulation of protein kinase C-α related MEK/ERK signaling pathways [16]. Antimicrobial activity of BV against skin bacteria in human monocytic cells was also found in our other study [17].

Our previous studies indicate that BV produces a fairly safe response against eukaryotic cell lines [9,10,17]. We found that BV did not affect the viability of eukaryotic cell lines such as human monocyte THP-1 cells, human dermal fibroblasts, and human epidermal keratinocytes at the concentrations tested. Under 1 μg/mL BV concentration, the BV had no significant effect either on the cell viability or morphological change. The findings of the present study revealed that BV has antimicrobial activities and synergistic effects with antibiotics against MRSA strains. The MICs for MRSA strains were determined using the microdilution technique. The MICs and MBCs were defined as the lowest concentration of an antimicrobial agent that prevented turbidity as assessed 24 h after inoculation. The obtained MICs and MBCs of BV indicate that BV is effective against MRSA strains. BV may be combined with other antimicrobial agents, such as ampicillin, penicillin, gentamicin and vancomycin, to increase the efficacy of the antimicrobial agents against methicillin-resistant organisms which provides a complementary natural antibacterial spectrum. Time-kill experiments, which are used to measure bacterial activity, appear to be more clinically relevant than the checkerboard technique [18]. Moreover, killing curves provide a dynamic picture of antimicrobial action and interactions over time, as opposed to the checkerboard, which is usually applied only once [19].

In the present study, the synergistic effect test was used to assess the activity based on the FIC indices and a cell diffusion assay. The FIC indices of BV in combination with ampicillin or penicillin ranged from 0.631 to 1.002 in MRSA CCARM 3366, and 0.772 to 1.002 in MRSA CCARM 3708. However, the bactericidal effect of BV against MRSA strains was obvious when it was combined with gentamicin or vancomycin. When the major bioactive compound possessing antibacterial activity from prenylated chalcone was investigated for its synergistic effect of the mixture of ampicillin or gentamicin against MRSA strains, the FIC indices ranged from 0.188 to 0.375 [2], indicating a better synergistic effect than BV despite of different usage of MRSA strains between two studies. In addition, the inhibition zone was increased considerably by the use of BV in combination with ampicillin or penicillin against MRSA strains (data not shown). Thus, evaluating studies of other antibiotic combinations with BV are important to ensure a complete evaluation of its efficacy. However, extra vigilance and caution need to be exercised at a time of data interpretation considering the newly suggested criteria of FIC indices [20]. RT-PCR demonstrated that BV enhanced the expression of *atl* average 20% compared with untreated MRSA. Although 20% increase seems modest, it implies the mediation of 20% more cell lysis. An effect of BV to interfere with the post-translational modification of murein hydrolase, thereby disrupting enzyme function could explain this apparent contradiction [21]. A similar conclusion was drawn regarding a strain of *S. aureus* that exhibited intermediate resistance to vancomycin, where zymographic analysis and microarray analysis revealed an increased expression of autolytic enzymes with a decreased autolysis in the phenotype. BV collected from the honeybee has a number of potential medicinal properties. Here, we report that BV has antibacterial and synergistic activities with ampicillin, penicillin, gentamicin or vancomycin against MRSA strains. Although these results cannot be directly applied to a clinical setting, the findings of the present study suggest that BV could be helpful for treating MRSA infection. The results of the present study are promising and may contribute to the use of natural products as drugs. More MRSA strains should be tested in further studies which need to evaluate the mechanisms of BV action on MRSA as well.

## 3. Experimental Section

### 3.1. General Information

#### 3.1.1. Bee Venom

Colonies of natural honey bees used in this study were maintained at the National Institute of Agricultural Science and Technology, Suwon, Korea. BV was collected using a BV collecting device (Chunggin, Korea) in a sterile manner under strict laboratory conditions. In brief, the BV collector was placed in the hive, and the bees were given enough electric shock to cause them to sting a glass plate from which dried BV was later scraped off. The collected BV was diluted in cold sterile water and then centrifuged at 10,000 *g* for 5 min at 4 °C to discard residues from the supernatant [10]. BV was lyophilized by a freeze dryer and refrigerated at 4 °C for later use. All of the bioactive components of BV used in the experiment were confirmed by size exclusion gel chromatography (AKTAexplorer, Pharmacia, Pleasanton, CA, USA) by dissolving in 0.1 M ammonium formate adjusted to pH 4.5. A Sephadex TM75 column (Amersham Biosciences, Piscataway, NJ, USA) with further purification by a Source 15RPC ST column (GE Healthcare, Little Chalfont, UK) with 0.1% trifluoracetic acid in 20% acetonitrile as the eluent was used to confirm the presence of melittin, the major active ingredient of BV.

#### 3.1.2. Bacterial Strains

The bacterial strains used were *S. aureus* CCARM 3366 (MRSA) and *S. aureus* CCARM 3708 (MRSA) from the Culture Collection of Antimicrobial Resistant Microbes (Seoul, Korea). Each strain has different spectrum of antibiotic resistance. All strains were grown aerobically at 37 °C in brain heart infusion (BHI; Difco Laboratories, Detroit, MI, USA) broth and agar.

### 3.2. Evaluation of Anti-Bacterial Activity

#### 3.2.1. Minimum Inhibitory Concentrations (MIC)

The MIC of BV was determined by the broth microdilution method in 96-well microtiter plates [22]. BV was dissolved in distilled water and then filtered through a membrane filter (0.2 μm pore size, Millipore, Billerica, MA, USA). Two-fold serial dilutions of BV were prepared in the appropriate broth media. MRSA (1 × 10^6^ colony forming units [CFU]/mL) was incubated with BV at two-fold serial dilutions in the appropriate broth media under anaerobic conditions for 24 h. The MIC was read as the lowest concentration of BV inhibiting visible growth of the test organism was determined (optically clear). No trailing was observed.

#### 3.2.2. Minimum Bactericidal Concentrations (MBC)

The MBC value was read as the lowest concentration of BV required for a 99.9% reduction in the viable MRSA cell population [23]. For determining MBC values, an aliquot (0.1 mL) of MIC mixtures that showed no growth was inoculated onto BHI plates and incubated at 37 °C for 48 h.

#### 3.2.3. Time-Kill Assays

Time-kill assays were performed using previously described standard CLSI guidelines [24]. Bacterial suspensions diluted with appropriate broth media to 1 × 10^8^ CFU/mL MRSA were pre-incubated at 37 °C. These samples were then co-incubated with BV adjusted to 1.0% in the appropriate broth media to give a final concentration of 2 × MIC and 10 × MIC. Aliquots of 100 μL of the culture before (0 h, positive control) and after (1, 4, 18 and 24 h) the addition of BV were used to estimate CFU on the appropriate agar plates with adequate dilution using buffered saline supplemented with 0.01% gelatin. Three plates were used for each sample and estimation of the CFU was repeated separately.

### 3.3. Synergistic Effects of BV on MRSA

The antibacterial effects of BV in combination with ampicillin, penicillin, gentamicin or vancomycin were assessed using the checkerboard test [25]. Fractional inhibitory concentration (FIC) indices were calculated using the following formula: FIC = (MICdrug A in combination/MICdrug A alone) + (MICdrug B in combination/MICdrug B alone). Briefly, bacterial cells (1 × 10^8^ CFU/mL) were inoculated into BHI and dispensed at 10 μL/well into 96 well microtiter plates. MICs were determined by serial two-fold dilutions of BV and/or antibiotics. After 16 h of incubation at 37 °C, minimum concentrations of BV that prevented the growth of test organisms were determined (defined as MICs). MIC values were determined using three independent assays and FIC indices were calculated from the FIC indices of BV and penicillin. FIC indices indicate synergy at values of no more than 0.5, partial synergy at values greater than 0.5 and less than 1, an additive effect for values of 1.0, indifferent effect for values greater than 1 and less than 4, and an antagonistic effect for values of 4.0 or greater [22].

### 3.4. RNA Isolation and RT-PCR

To determine the effect of BV on expression of the *atl* gene which encodes murein hydrolases, MRSA was grown in BHI with and without 100 ng/mL BV at 37 °C for 4 h. The decision on BV dose was made following a preliminary test. RNA was isolated using the QIAGEN RNeasy^®^ Mini Kit and RNA protect^®^ Bacteria Reagent (Qiagen, Valencia, CA, USA). Primers for *atl* and *ygil* (a housekeeping gene that codes for acetyl coenzyme A) were designed using NCBIPrimer-BLAST to be 20 to 24 bases long to have a GC content of more than 50%, a melting temperature of approximately 60 °C, and to amplify PCR products of 137 bp and 243 bp for *ygil* and *atl*, respectively [26]. The primers (Genotec, Daejeon, Korea) utilized for RT-PCR were as follows: *ygil* (forward: 5’-GAC GTG CCA GCC TAT GAT TT-3’, reverse: 5’-ATT CGT GCT GGA TTT TGT CC-3’); *atl* (forward: 5’-AAT CAA GGT GGC ACA CAA CA-3’, reverse: 5’-CTGGATGCTCATATTGACG-3’). All reactions were performed in triplicate and *atl* expression was analysed relative to the expression of the housekeeping gene. The PCR products were analyzed by 2% agarose gel electrophoresis with ethidium bromide. The signal intensity of each band was quantified and normalized against GAPDH. Densitometric analysis was measured using Quantity One (Bio-Rad, Hercules, CA, USA) to scan the signals.

### 3.5. Statistical Analysis

All data are expressed as the mean ± standard error of the mean (SEM). Statistical differences among groups were calculated by analysis of variance (ANOVA) followed by Duncan’s multiple range test (SPSS 18.0 version., Chicago, IL, USA). Differences with *p* < 0.05 were considered significant.

## 4. Conclusions

We reported that BV collected from the honeybee has antibacterial and synergistic activities with ampicillin, penicillin, gentamicin or vancomycin against MRSA strains. The antimicrobial activity of BV against MRSA strains was investigated using MIC, MBC and a time-kill assay. Moderately high BV MBCs relative to the MIC were shown which indicates that BV inhibited the growth of these MRSA strains at relatively low concentrations. The bactericidal activity of BV corresponded to a decrease of CFU. The combination of BV with ampicillin, penicillin yielded a partial and indifferent synergistic effect. However, gentamicin or vancomycin enhanced the bactericidal activity of BV against MRSA strains. The exposure of MRSA to BV caused an increased expression of *atl* gene. BV warrants further investigation as a natural antimicrobial agent and synergist of antibiotic activity.
